# RVX-208, an Inducer of ApoA-I in Humans, Is a BET Bromodomain Antagonist

**DOI:** 10.1371/journal.pone.0083190

**Published:** 2013-12-31

**Authors:** Kevin G. McLure, Emily M. Gesner, Laura Tsujikawa, Olesya A. Kharenko, Sarah Attwell, Eric Campeau, Sylwia Wasiak, Adam Stein, Andre White, Eric Fontano, Robert K. Suto, Norman C. W. Wong, Gregory S. Wagner, Henrik C. Hansen, Peter R. Young

**Affiliations:** 1 Resverlogix Corp., Calgary, Alberta, Canada, or San Francisco, California, United States of America; 2 Xtal BioStructures Inc., Natick, Maryland, United States of America; Institute of Enzymology of the Hungarian Academy of Science, Hungary

## Abstract

Increased synthesis of Apolipoprotein A-I (ApoA-I) and HDL is believed to provide a new approach to treating atherosclerosis through the stimulation of reverse cholesterol transport. RVX-208 increases the production of ApoA-I in hepatocytes *in vitro*, and *in vivo* in monkeys and humans, which results in increased HDL-C, but the molecular target was not previously reported. Using binding assays and X-ray crystallography, we now show that RVX-208 selectively binds to bromodomains of the BET (Bromodomain and Extra Terminal) family, competing for a site bound by the endogenous ligand, acetylated lysine, and that this accounts for its pharmacological activity. siRNA experiments further suggest that induction of ApoA-I mRNA is mediated by BET family member BRD4. These data indicate that RVX-208 increases ApoA-I production through an epigenetic mechanism and suggests that BET inhibition may be a promising new approach to the treatment of atherosclerosis.

## Introduction

Atherosclerosis continues to be a major cause of morbidity and mortality despite significant reductions in coronary events through the use of statins, which inhibit HMGCoA reductase and intracellular cholesterol synthesis. These drugs significantly lower LDL-C and can arrest plaque accumulation, but prevent only about 30–40% of cardiovascular events. In contrast to the linear correlation between decreases in low-density lipoprotein cholesterol (LDL-C) and cardiovascular risk [1], [2] such that lowering LDL-C reduces CVD events, high density lipoprotein cholesterol (HDL-C) levels correlate inversely with CVD risk [3]. However, raising the levels of HDL does not always lower CVD risk [4], [5], [6]. This may arise from considerable heterogeneity in the size, shape, composition and function of different HDL particles [7], [8], [9], and in particular their ability to mediate reverse cholesterol transport, the process by which excess cholesterol in plaque is removed from the body via uptake and excretion from the liver. Hence the focus of current HDL elevating therapies is to increase functional HDL.

While there are several approaches to the therapeutic modification of HDL currently in clinical development [10] including the modification of factors involved in HDL metabolism and remodeling [11], [12], [13], [14], the most direct test of whether increasing functional HDL is a viable approach has been provided by clinical studies in which HDL is infused into patients [15], [16], [17]. In these landmark studies, a significant regression of coronary plaque was found to result from HDL infusion after just a few weeks, suggesting that one way to increase functional HDL is through de novo synthesis.

RVX-208 is a small molecule undergoing clinical development as a potential therapy to enhance ApoA-I production [18], [19] and hence treat atherosclerosis and prevent CVD events. RVX-208 increases ApoA-I expression in liver cells *in vitro*, and *in vivo* studies in monkeys have demonstrated that ApoA-I induction with RVX-208 leads to enhanced systemic capacity to promote cholesterol efflux [18]. More recently, early clinical trials of RVX-208 in statin-treated patients with coronary artery disease demonstrated increased ApoA-I and HDL-C levels [19]. RVX-208 is now being clinically evaluated for its ability to regress plaque in the coronary arteries [20]. However, because RVX-208 was discovered in a phenotypic screen for compounds that enhanced ApoA-I mRNA expression in a human hepatocarcinoma cell line, its molecular target was not initially known.

In this report, we identify the molecular target of RVX-208 to be the BET proteins, and in particular the BET family member BRD4, which regulates ApoA-I expression through an epigenetic mechanism.

## Materials and Methods

### Chemical synthesis

RVX-208 (2-(4-(2-hydroxyethoxy)-3,5-dimethylphenyl)-5,7-dimethoxyquinazolin-4(3H)-one) was synthesized by NAEJA Pharmaceuticals (Edmonton, Canada) and IRIX Pharmaceuticals (Florence, South Carolina, USA) to support both non-clinical and clinical work. Synthetic procedures can be found in US Patents 8,114,995 [21] and 8,053,440 [22]. Melting point (uncorrected): 231–233°C; MS (EI+): 371.11 (M+H^+^); ^1^H-NMR (DMSO-d_6_): δ 11.8 (s, 1H), 7.9 (s, 2H), 6.8 (br s, 1H), 6.6 (br s, 1H), 4.9 (br s, 1H), 3.7–3.9 (m, 10 H), 2.3 (s, 6H); ^13^C-NMR (DMSO-d_6_): δ 164.9, 161.7, 160.4, 159.1, 153.8, 153.2, 131.5, 128.9, 127.9, 105.3, 101.8, 98.2, 74.7, 61.1, 56.6, 56.3, 16.8. JQ-1 was synthesized as described [23]and characterized by 1H-NMR, Mass spectroscopy, HPLC (UV), and chiral HPLC (UV). The generated data was in agreement with published values. The calculated enantiomeric excess (%ee) was 70%ee.

### Cell culture

Huh7 cells were plated at 23,000/well in a 96 well plate in DMEM +10% FBS before allowing to grow overnight. Cells were treated with compounds for 48 h in 0.1%DMSO with or without 5 µM Actinomycin D. U937 cells were differentiated for 3 days in 60 ng/mL PMA, 32,000 cells/well in 96-well format. Cells were then treated with compound in 0.1%DMSO in RPMI media +10%FBS, and after 1 h, lipopolysaccharide (LPS, Sigma) was added to the cells at 1 µg/mL for 3 hours.

### RT-PCR

Cells were harvested by mRNA Catcher PLUS Kit followed by real-time PCR using the RNA UltraSense One-Step qRT-PCR System. ApoA-I, IL-6 and TNFα mRNA levels were measured relative to the endogenous control serpin A1 or cyclophilin in the same sample. Data was acquired using the 7500 Real Time PCR System (Applied Biosystems).

### Bromodomain expression

Single bromodomain and dual domain BRD4[BD1BD2] constructs with an *N*-terminal His-tag based on [23], [24]were cloned, expressed, and purified by Nickel affinity and size-exclusion chromatography by either Genscript or Xtal BioStructures, Inc. Fractions representing monomeric protein were pooled and frozen at −80°C for use in subsequent experiments.

### Protein Thermal Denaturation Assay

5 µM of purified bromodomain protein was incubated with 5X SYPRO® Orange (Molecular Probes) at a final concentration of 20 mM HEPES pH 7.4, 100 mM NaCl in the presence of 100 µM compound or DMSO (0.2%) in a fast 96 well optical plate (Applied Biosytems). Samples were incubated at room temperature for 30 minutes and ramped from 25°C to 95°C in a ViiA7 real-time PCR machine (Applied Biosystems). The resulting fluorescence data was analyzed and melting temperatures calculated using Protein Thermal Shift™ Software v1.0 (Life Technologies).

### Time Resolved Fluorescence Resonance Energy Transfer (TR-FRET) assay

200 nM *N*-terminally His-tagged bromodomains or BRD4(BD1BD2) and 25–50 nM biotinylated tetra-acetylated histone H4 peptide (Millipore) were incubated in the presence of Europium Cryptate-labeled streptavidin (Cisbio Cat. #610SAKLB) and XL665-labeled monoclonal anti-His antibody (Cisbio Cat.#61HISXLB) in a white 96 well microtiter plate (Greiner). For inhibition assays, serially diluted compound was added to these reactions in a 0.2% final concentration of DMSO. Final buffer concentrations were 30 mM HEPES pH 7.4, 30 mM NaCl, 0.3 mM CHAPS, 20 mM PO_4_ pH 7.0, 320 mM KF, 0.08% BSA). After 2 h incubation at room temperature, the fluorescence by FRET was measured at 665 and 620 nm by a SynergyH4 plate reader (Biotek). IC_50_ values were determined from a dose response curve.

### Isothermal Calorimetry

150 µM BRD2[BD1], BRD2[BD2], BRD4[BD1] or BRD4[BD2]in 50 mM HEPES pH 7.5, 150 mM sodium chloride and 0.05% DMSO was injected into a solution containing 10 µM RVX-208 in the same buffer at 25°C, and the associated change in heat measured in a Microcal Auto-ITC instrument.

### Extraction of soluble BET proteins from chromatin

Soluble proteins were extracted using a buffer containing 0.5% Triton X-100 (10 mM PIPES pH 6.8, 300 mM sucrose, 50 mMNaCl, 3 mM MgCl_2_, 1 mM EGTA, 2 mM ribonucleoside vanadyl complex) as previously described [25]. After loading on a 4–12% NuPAGE gradient gel (Life Technologies), the soluble and chromatin-containing pellet fractions were evaluated by Western blot with the following antibodies and dilutions: BRD2 (Bethyl, A302-583A, 1∶5000); BRD3 (Bethyl A302-368A, 1∶2000); BRD4 (Bethyl A301-985A, 1∶1000); PHIP (Abcam ab86244, 1∶2000), HNF4a (Perseus PPH1415, 1∶1000); NR2F2 (Perseus PPH7147, 1∶1000).

### siRNA

Combinations of siRNAs or individual siRNAs were used in a single or double transfection protocol to achieve efficient mRNA and protein knockdown (at least 60% at the protein level). All siRNAs were obtained from Dharmacon (Brd2 L-004935-00-0005 and LU-004935-00-0002; Brd3 L-004936-00-0005; Brd4 L-004937-00-0005 and LU-004937-00-0002; OnTARGET Plus control siRNA). The siRNA was prepared as per manufacturer's instructions (Dharmacon) and incubated with 23,000 cells/well Huh-7 cells in DMEM containing 10% FBS at a final concentration of 100 nM. 48 h post-transfection siRNA-containing media was replaced with fresh media containing 0.1% DMSO and incubated for a further 48 h. To track BRD expression, proteins were collected in 20 µL/well of 10 mM Hepes pH 7.4, 2% SDS, protease inhibitors' cocktail (Life Technologies) on treatment day or at the end of the experiment. mRNA was collected at 3 h and 48 h post transfection. In a double transfection, a second round of siRNA was added after the first 48 h siRNA incubation, and 24 h later was replaced with fresh medium containing 0.1% DMSO for a further 48 h.

### X-ray Crystallography

The recombinant human BRD4[BD1] and human BRD2[BD2] protein domain constructs were each concentrated to 10 mg/mL in a buffer consisting of 10 mM HEPES pH 7.5, 150 mM NaCl, 0.5 mM TCEP. The compound RVX-208 was dissolved to 50 mM in DMSO and then added to the proteins to obtain a final 2 mM concentration; gently mixed; and incubated on ice for 30 minutes. Sparse matrix crystallization screens were evaluated at room temperature and yielded crystals for BRD4[BD1]/RVX-208 with the solution of 25% PEG 3350 (w/v), 0.1 M Bis-Tris pH 6.5, and 0.2 M Li_2_SO_4_, and for BRD2[BD2]/RVX-208 with a solution of 25% PEG1500 (w/v). Selected monocrystals were quickly transferred into a cryo-protectant solution containing the well solution supplemented with 10% (v/v) ethylene glycol; a single crystal was harvested with a nylon loop; flash-cooled into liquid nitrogen; and stored at cryogenic temperature for later analyses at the synchrotron.

Single-crystal X-ray diffraction was obtained at Beam line X29 of the National Synchrotron Light Source, Brookhaven National Laboratory, using an automated sample mount system. The X-ray diffraction data were reduced using *HKL2000* [26]. The BRD4[BD1] crystal diffracted to 1.24 Å resolution and indexed with a primitive orthorhombic unit cell having the dimensions of *a* = 37.278 Å, *b* = 44.623 Å, and *c* = 78.064 Å. The space group was determined to be P2_1_2_1_2_1_on the basis of both the systematic absences and the more favorable χ^2^ values of high ordered symmetry. The calculated Matthews coefficient of V_m_ = 2.4 Å^3^/Da suggests a solvent content of 49%,given one molecule of BRD4[BD1]/RVX-208 complex in the asymmetric unit. The crystal of BRD2[BD2] diffracted to 1.08 Å resolution and indexed with a *C*-centered orthorhombic unit cell having the dimensions of *a* = 51.100 Å, *b* = 66.350 Å, and *c* = 75.854 Å. The space group was determined to be C222_1_on the basis of the systematic absences. The calculated Matthews Coefficient of V_m_ = 2.4 Å^3^/Da suggests a solvent content of 48%, given one molecule of BRD2[BD2]/RVX-208 complex in the asymmetric unit. The final crystallographic data reduction statistics are summarized in Table 1.

**Table 1:** X-ray data reduction statistics and crystal parameters.

	hBRD4(BD1)/RVX-208	hBRD2(BD2)/RVX-208
Space group	P2_1_2_1_2_1_	C222_1_
Unit cell(Å)	*a* = 37.278, *b* = 44.623, *c* = 78.064	*a* = 51.100, *b* = 66.350, *c* = 75.854
Resolution range (Å)	50–1.24	50–1.08
Number of measurements	209,679	432,621
Number of unique reflections	37,571	55,428
*R*_sym_(%)^a^	0.053 (0.24)	0.043 (0.39)
Completeness (%)^a^	99.7 (100.0)	99.6 (99.9)
*I*/σ^a^	24.2 (4.9)	37.3 (4.2)
Redundancy^a^	3.9 (3.8)	4.6 (4.5)
Mosaicity (°)	0.131	0.378

^a^ Numbers in the parentheses are for the highest resolution shell of 1.28-1.24 Å for BRD4[BD1]/RVX-208) and 1.12-1.08 Å for BRD2[BD2]/RVX-208.

The structures were solved by molecular replacement with data from 10-3.0 Å resolution using PHASER (McCoy, 2007). The BRD4 structure (RCSB entry 3MXF) [23]was used as a starting model to solve the complex of BRD4[BD1]/RVX-208. Similarly, the BRD2 structure (RCSB entry 3ONI) [23]was used as a starting model to solve the complex of BRD2[BD2]/RVX-208.The solution of each of these two structures was then refined using both rigid body and positional refinementwithREFMAC5 [27] to an *R*/*R*_free_ of 23%/25% for BRD4[BD1] and to 23%/26%for BRD2[BD2]. Inspection of the initial electron density maps showed unambiguous density for the compound RVX-208 in each of the two structures. Several rounds of model building and restrained refinement were performed in the absence of RVX-208. Solvent molecules were then positioned in each of the two structures in order to improve the clarity of the residual element of density corresponding to the ligand. The RVX-208 compound and some solute molecules were then included with the structure and refined. The RVX-208 compound bound to BRD4[BD1] with two alternate conformers for the di-methyl phenoxy ethyl alcohol moiety. However, binding of RVX-208 to BRD2[BD2] showed only one stable binding mode. Given the high resolution of these two structures, individual anisotropic temperature factors were also refined. The BRD4[BD1]/RVX-208 structure refined to *R*/*R*_free_ of 11.0%/14.5% using the data extending to 1.24 Å resolution; the BRD2[BD2]/RVX-208 structure refined to *R*/*R*_free_ of 12.7%/15.9% at 1.08 Å resolution. The final refinement statistics are listed in Table 2. The structures have been deposited in the PDB with the following codes: 4J1P and 4J3I.

**Table 2:** Crystallographic refinement statistics.

	hBRD4(BD1)/RVX-208	hBRD2(BD2)/RVX-208
Resolution range (Å)	20–1.24	20–1.08
*R*_cryst_ (%)	11.3	12.8
*R*_free_ (%)	14.5	16.0
Number of ligand/EDO/waters	1/2/257	1/0/255
Rmsd bond lengths (Å)^a^	0.025	0.027
Rmsd bond angles (°)^a^	2.21	2.40
Average *B*-factors (Å^2^)		
Main chain atoms	7.14	10.72
Side chain atoms	8.95	12.78
RVX- 208	10.40	11.9
EDO	11.69	N/A
Waters	17.75	22.46
Ramachandran Plot (%)		
Favored	97.4	100
Generously allowed	2.6	0
Disallowed	0	0

^a^ **Root-mean square deviation (rmsd) from the standard stereochemistry** [**52**] **.**

## Results

### Transcriptional regulation of ApoA-I by RVX-208

**Figure 1:**
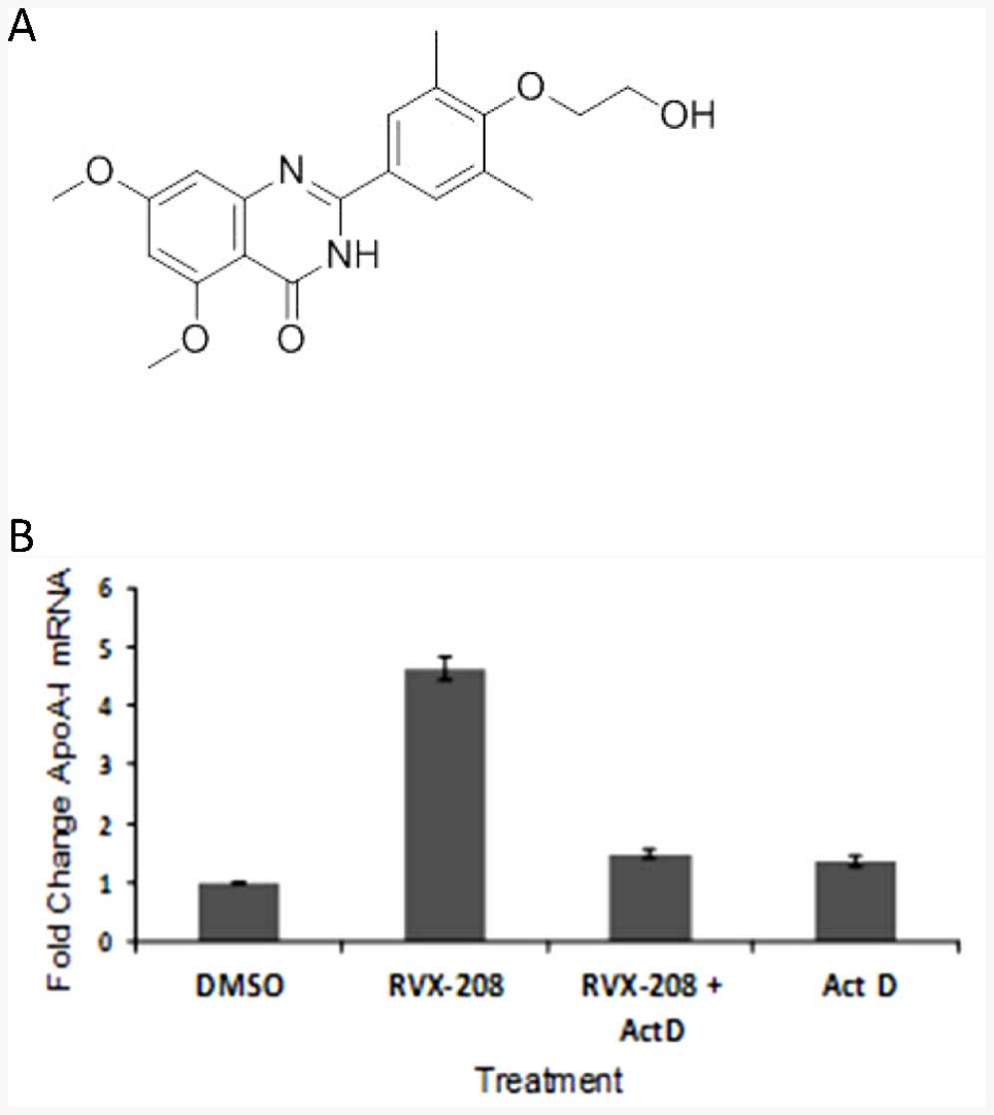
Structure of RVX-208 and dependence of RVX-208 induced ApoA-I on transcription. A. Chemical structure of RVX-208. B. Induction of ApoA-I in the human hepatoma cell line Huh7 by 30 µM RVX-208 is inhibited by Actinomycin D (Act D).

RVX-208, which is a quinazolin-4-one derivative (Figure 1A), induces ApoA-I mRNA in human hepatocyte cell lines, such as HepG2 [18] and Huh7 (Figure 1B). Induction is inhibited by actinomycin D, a known inhibitor of transcription, indicating that RVX-208 regulates ApoA-I through a transcriptional mechanism (Figure 1B).

**Figure 2:**
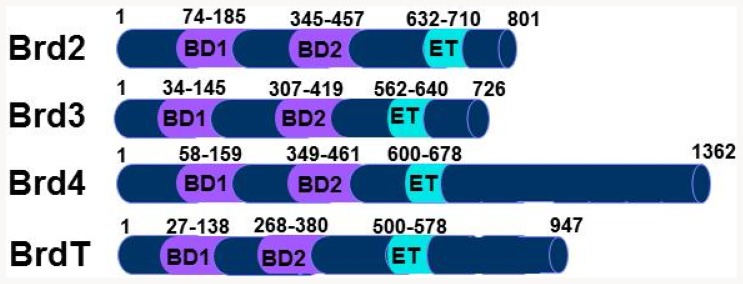
Schematic of domain structure of the human BET proteins. Bromdomains are designated by BD1 and BD2 and the Extraterminal domains by ET.

**Figure 3:**
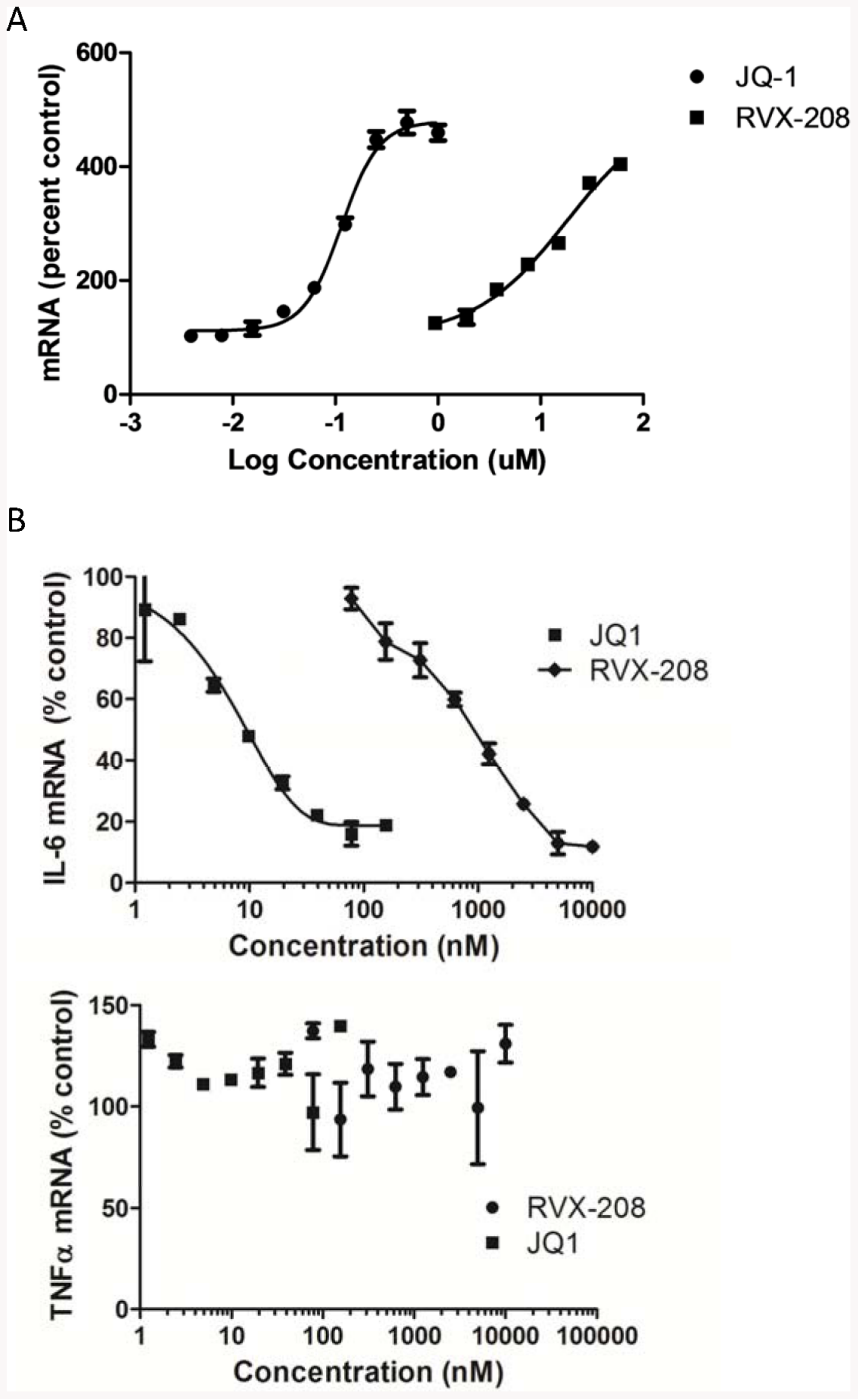
Stimulation of ApoA-I and inhibition of IL-6 production by RVX-208 and JQ-1. A. Dose dependent induction of ApoA-I in Huh7 cells by RVX-208 and JQ-1 at 48 h, B. Selective dose dependent inhibition of IL-6 but not TNFα mRNA in U937 cells stimulated with LPS for 4 h.

An epigenetic mechanism for regulating transcription involving the BET proteins was recently described for small molecules with a benzodiazepine core structure, JQ-1 and I-BET, distinct from RVX-208 [23], [24]. The BET protein family includes four proteins, BRD2, BRD3, BRD4 and BRDT, that bind to select acetylated lysine residues on histones and other proteins via two tandem ∼110 amino acid bromodomains, and thereby recruit additional proteins that regulate transcription [28] (Figure 2). JQ-1 competes for the acetylated lysine binding site within the BET bromodomains, leading to dissociation of BET proteins from chromatin and regulation of transcription. Like RVX-208, JQ-1 induces ApoA-I mRNA in Huh7 cells (Figure 3A) and selectively suppresses IL-6 mRNA, but not TNFα, in a human macrophage-like cell line (Figure 3B) [24], suggesting that they might work through a similar mechanism.

### Binding of RVX-208 to BET bromodomains

To explore the potential role of the BET proteins in RVX-208 activity, we expressed and purified the bromodomains (BD1, BD2) for the BETs expressed in hepatocytes (BRD2, BRD3, BRD4) and evaluated binding of RVX-208 by thermal denaturation. In this technique, the increase in the T_m_ for BET bromodomain denaturation in the presence of ligand is proportional to its affinity [23]. Like JQ-1, RVX-208 increased the T_m_ of all BET bromodomains, indicative of binding (Table 3 and Figure S1). However, RVX-208 did not bind to bromodomains contained within six other proteins (CREBBP, p300, BAZ2B, GCN5, PHIP and SMARC2A) suggesting that RVX-208 binding, like JQ-1, is selective for BET protein bromodomains.

**Table 3:** Binding of RVX-208 to individual bromodomains.

Bromodomain	Thermal denaturation ΔT_m_ (°C)		TR-FRET IC50 (µM)	
	RVX-208	JQ-1	RVX-208	JQ-1
BRD2[BD1]	1.9	6.7	2.6	0.09
BRD2[BD2]	5.1	7.7	0.09	0.01
BRD3[BD1]	2.6	9.6	3.1	0.04
BRD3[BD2]	6.8	9.0	0.28	0.03
BRD4[BD1]	3.9	10.3	1.8	0.12
BRD4[BD2]	7.7	5.5	0.04	0.01
CREBBP	−0.3	−1.0	-	-
BAZ2B	−0.0	−0.83	-	-
P300	−0.16	−1.4	-	-
PHIP	0.19	−1.4	-	-
GCN5	0.0	−0.7	-	-
SMARCA2A	0.4	0.4		

**Figure 4:**
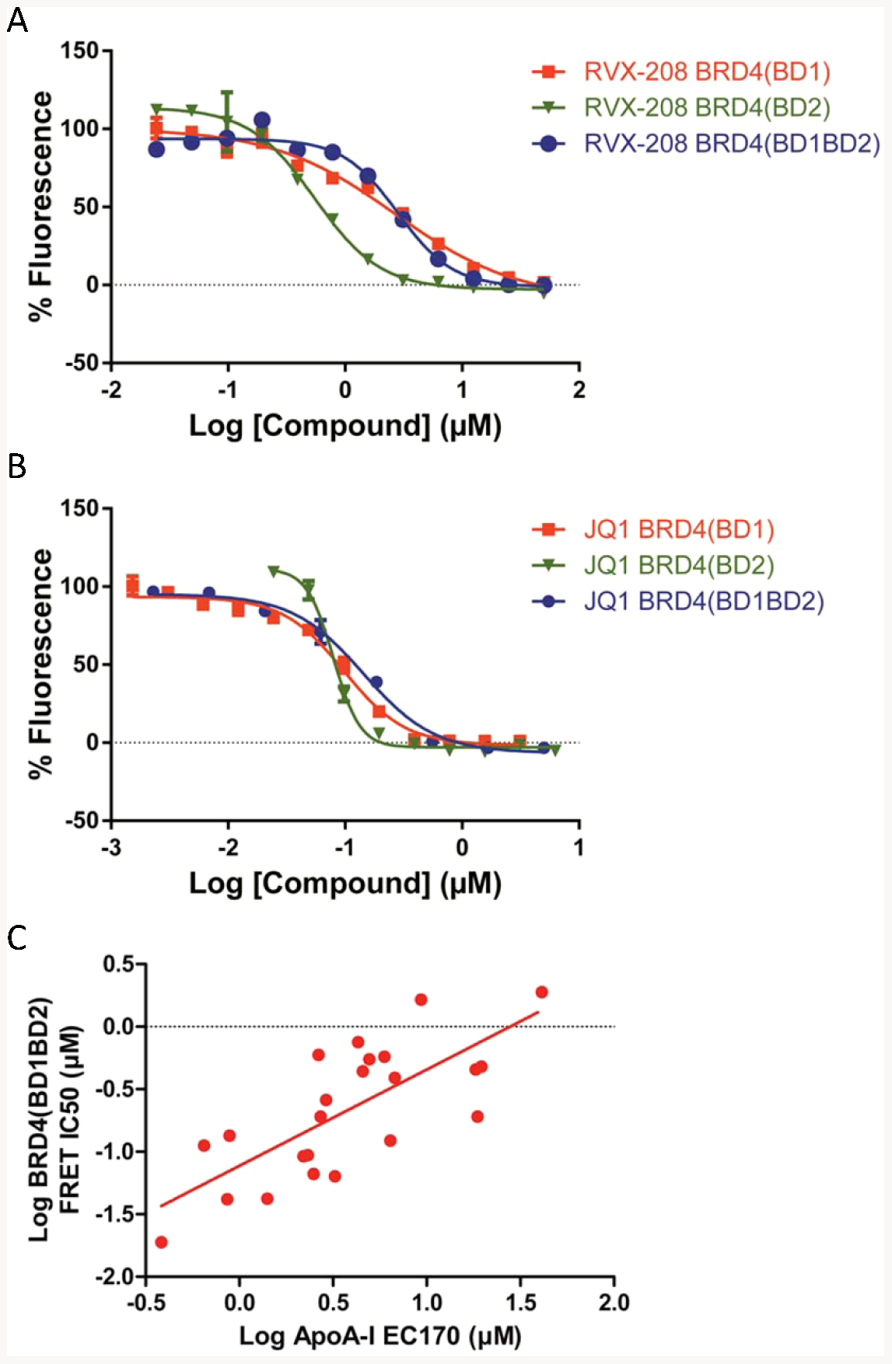
Selective binding of RVX-208 to bromodomains of the BET family. A. Competition of RVX-208 for binding to BRD4 bromodomains 1 and 2 individually or in tandem as measured in a competition FRET assay with acetylated histone 4 peptide. B. Same as A except for JQ-1. C. TR-FRET IC_50_ for binding of 23 RVX-208 analogues to Brd4 [BD1BD2] is plotted against EC170 for induction of ApoA-I mRNA in Huh7 cells.

In the cell, bromodomains recognize acetylated lysine in the context of specific peptide sequences as found, for example, at the amino terminus of histones. To evaluate if the binding of RVX-208 leads to the displacement of acetylated peptide binding to BET bromodomains, we established a competition TR-FRET assay between individual His-tagged bromodomains and an acetylated peptide derived from the amino terminus of histone H4. RVX-208 competes for acetylated histone H4 peptide binding to both bromodomains of BRD4, similar to JQ-1, but with a preference for BD2 over BD1 (Figure 4A, B and Table 3). RVX-208 also binds to the bromodomains of BRDs 2 and 3 with a similar preference for BD2 (K_D_∼5–30 nM) over BD1 (K_D_∼2–3 µM) (Table 3). JQ-1 does not show such a preference. RVX-208, like JQ-1, also competes with binding of an acetylated histone peptide to tandem BD1 BD2 protein constructs of the four BET proteins, with IC50s between 0.5 and 1.8 µM (Table 4).

**Table 4:** Binding of RVX-208 to tandem bromodomains [BD1 BD2].

Bromodomain	TR-FRET IC50 (µM)	
	RVX-208	JQ-1
BRD2	1.81	0.11
BRD3	0.78	0.04
BRD4	0.58	0.08
BRDT	1.45	0.12

To determine the pharmacological relationship between RVX-208 binding and ApoA-I induction, we evaluated 254 compounds structurally related to RVX-208 for their binding to BRD4[BD1]and ability to induce ApoA-I mRNA in Huh7 cells. 180 (71%) compounds were positive and 45 (18%) were negative in both assays, supporting a requirement for BET bromodomain binding for induction ApoA-I mRNA. In contrast 26 (10%) compounds bound to BRD4[BD1] but did not induce ApoA-I, consistent with an inability to accumulate in cells, whereas only 3 (1%) compounds demonstrated induction of ApoA-I in the absence of BRD4[BD1] binding, which could be attributed to aberrant data or the possibility that these compounds worked by an alternate mechanism.

To further explore this structure-activity relationship, we determined the IC50s of multiple compounds for binding to a fragment of BRD4 containing both BD1 and BD2. Comparison with their EC170s for induction of ApoA-I mRNA in Huh7 cells (EC170 is the dose required to give a 70% increase in ApoA-I mRNA) (Figure 4C) indicated a linear relationship, supporting a pharmacological relationship between the two, and further supporting the role of BET bromodomain binding in driving ApoA-I induction.

### Displacement of BET proteins from chromatin by RVX-208

**Figure 5:**
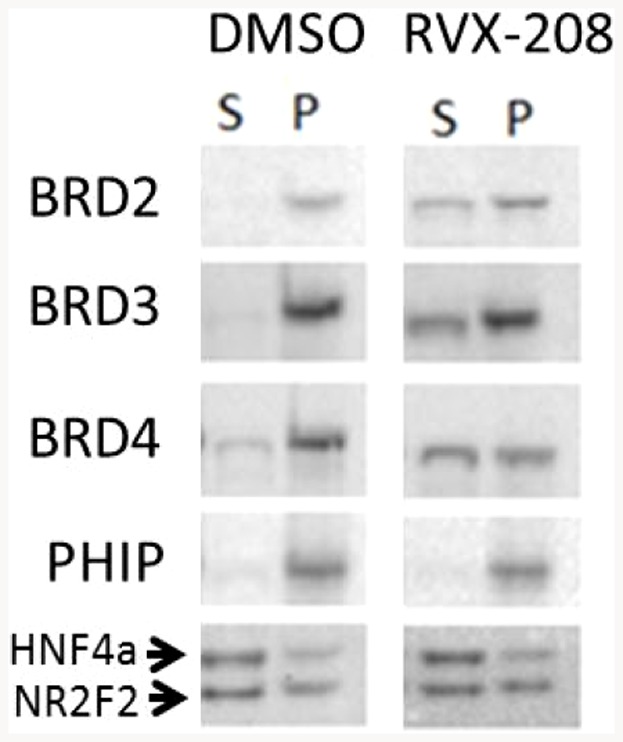
Selective release of BET proteins from chromatin in Huh7 cells by RVX-208. Salt extraction of chromatin releases BET proteins into the soluble (S) fraction in RVX-208 stimulated cells, whereas another dual bromodomain protein PHIP stays in the insoluble (pellet, P) fraction. The solubility of other transcription factors (HNF4a, NR2F2) is unaltered.

If the displacement of acetylated histone peptide regions from BET bromodomains by RVX-208 is relevant to gene regulation, we would expect to see displacement of BET proteins from chromatin in cells. When Huh7 cells are incubated with RVX-208 or JQ-1, BRDs2, 3 and 4 are partially released from chromatin as indicated by the increase in these proteins in the soluble fraction of suitably prepared lysates (Figure 5). This occurs within 2 h of treatment and is maintained for more than 48 h. The release was selective for the BET proteins and was not seen with PHIP, another bromodomain containing protein, or other transcription factors, consistent with the in vitro findings. Significant amounts of the BET proteins remained bound to chromatin, likely through domains distinct from the bromodomains.

### Crystal structures of complexes of RVX-208 with BRD4[BD1] and BRD2[BD2]

**Figure 6:**
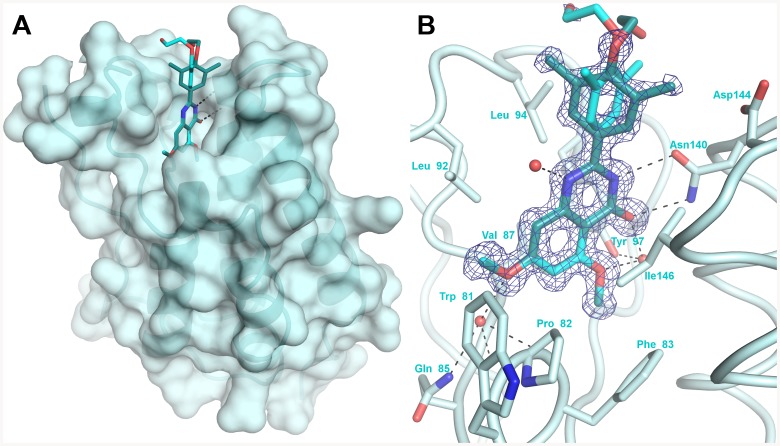
X-ray crystal structure of RVX-208 bound to BRD4[BD1]. A. Surface rendering model showing disposition of RVX-208 in hydrophobic pocket. B. A closer view of the BRD4[BD1] ligand binding site. The electron density (sigmaA-weighted, 1σ contour level, 1.24 Å resolution) corresponding to the compound is shown in blue. For clarity, only interacting residues and some water molecules are shown with a ribbon diagram of the protein main chain course.

To determine how RVX-208 binds to bromodomains, we selected two purified bromodomains: BD1 of BRD4 and BD2 of BRD2. Within the BET family the BD1 bromodomains are more homologous to each other than they are to the BD2 domains and similarly for the BD2 domains. A 1.24 Å resolution crystal structure of RVX-208 bound to BRD4[BD1] was obtained (Figure 6). BRD4[BD1] exhibits the conserved, left handed, α-helical bundle consisting of four alpha helices (α_Z_, α_A_, α_B_, α_C_) and two interconnecting loops (ZA and BC), and is virtually identical to the published structure of BRD4 with JQ-1 (RCSB entry 3MXF) with an RMSD of 0.111 Å. RVX-208 binds with the plane of the quinazolin-4-one oriented parallel to the alpha helices that make up the bromodomain with the dimethyl-phenyl region of RVX-208 existing in two distinct conformations resulting from rotation around the carbon-carbon linker with the quinazolin-4-one. RVX-208 binds BRD4[BD1] in a mostly hydrophobic pocket where it is flanked by Val87, Leu92 and Leu94 side chains on one side of the compound and Pro82 and Leu146 side chain on the other. It is also within van der Waals distances to the four aromatic residues Tyr97, Tyr139, Phe83 and Trp81. RVX-208 quinazolin-4-one also forms a bidentate set of two hydrogen bond interactions with the conserved Asn140 amide side chain, the amino acid recognized by acetylated lysine of the natural substrate, as well as an extensive water network. The quinazolin-4-one scaffold ketone and methoxy oxygens both form a water-mediated interaction with the Tyr97 hydroxyl group. The other compound methoxy oxygen also forms a water-mediated interaction with Gln85. Several of the amino acids in the binding pocket, Trp81-Phe83, Asn140, Val87, Leu92, and Leu94 are conserved among all bromodomains.

**Figure 7:**
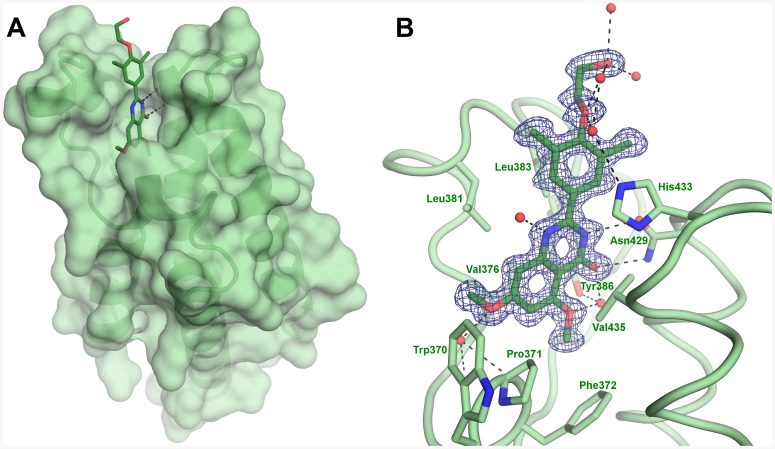
X-ray crystal structure of RVX-208 bound to BRD2[BD2]. A. Surface rendering model showing disposition of RVX-208 in hydrophobic pocket. B. A closer view of the BRD2[BD2] ligand binding site. The electron density (sigmaA-weighted, 1σ contour level, 1.08 Å resolution) corresponding to the compound is shown in blue. For clarity, only interacting residues and some water molecules are shown with a ribbon diagram of the protein main chain course. The waters were removed for clarity.

To assess the binding to BD2, we also determined the X-ray crystal structure of RVX-208 bound to BD2 of BRD2 to 1.08 Å resolution (Figure 7). RVX-208 adopts a very similar binding configuration as seen with BRD4[BD1], although the dimethyl-phenyl group is now fixed in one conformation. The quinazolin-4-one is flanked by Val376, Leu381 and Leu383 side chains on one side of the compound and Pro480 and Val435 on the other. It is also within van der Waals distances to the four aromatic residues Tyr386, Phe372, Trp370 and His433. When compared to the structure of BRD2[BD2] with JQ-1 (RCSB entry 3ONI), BRD2[BD2]: RVX-208 is virtually identical with an RMSD of 0.269 Å. Once again the binding is driven by hydrophobic interactions, a bidentate set of two hydrogen bond interactions with Asn429, the amino acid recognized by the natural substrate, a water-mediated interaction between the quinazolin-4-one ketone and methoxy oxygens with the Tyr386 hydroxyl group, a water-mediated H-bond between the compound methoxy and Lys374 carbonyl oxygen and a network of hydrogen bonds to bound water molecules.

**Figure 8:**
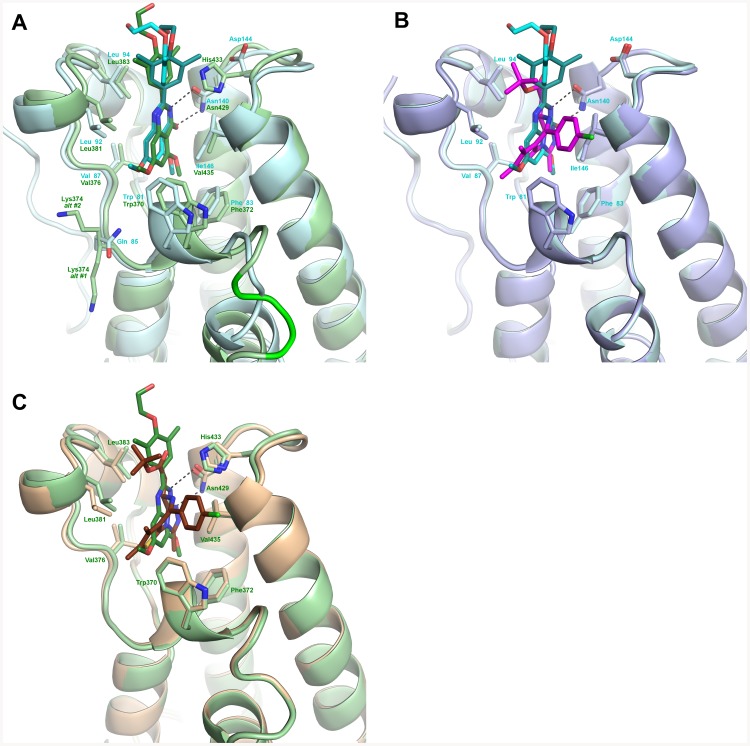
Comparison of RVX-208 and JQ-1 interactions with the bromodomain ligand binding sites. A. Overlay of BRD4[BD1] (pale green, cyan and light gray) and BRD2[BD2] (green and wheat) when complexed with RVX-208. The three residue insertion difference in BRD2[BD2] is colored in yellow. In all three panels, the hydrogen bonds are illustrated with dashed lines and the waters are removed for clarity. B. A comparison of the BRD4[BD1] when complexed with RVX-208 (pale green, cyan and light gray) and with JQ-1(purple and light blue, 3MXF.pdb). C. A comparison of the BRD2[BD2] when complexed with RVX-208 (green and wheat) and with JQ-1 (burgundy and salmon, 3ONI.pdb).

Several of the residues surrounding the RVX-208 binding pocket in BRD2[BD2], Trp370-Phe372, Asn429, Val376, Leu381, and Leu383 are shared with BET BD1 domains (Figure 8A). However Asp144 and Ile146, which are conserved amongst all BET BD1 domains, are replaced by His433 and Val435 respectively in BRD2[BD2] and all other BET BD2 domains. This could contribute to the BD1 versus BD2 selectivity of RVX-208 and to the finding that RVX-208 bound to BRD2[BD2] adopts only one of the conformations in BRD4[BD1]. Whereas Asp144 points away from the ligand binding in BRD4[BD1] complexes, the homologous His433 found in BRD2[BD2] forms van der Waals contacts with RVX-208 and thus does not allow multiple rotational conformers of the dimethyl-phenyl group. In both of these bromodomain structures, the ethanol alkyl chain of RVX-208 does not form stable direct interaction with the protein.

RVX-208 binds to BRD4[BD1] and BRD2[BD2] quite differently than does JQ-1, which is based on a triazolodiazepine core scaffold (Figure 8B, C). RVX-208 lacks the interaction with the “WPF” motif seen with the phenyl chloride moiety of JQ-1 and the H-bond with His433 in BRD2[BD2] provided by the external ester of JQ-1. However, RVX-208 forms a bidentate interaction with the conserved Asn140 of BRD4[BD1] and Asn429 of BRD2[BD2], while JQ-1 forms only a single H-bond interaction. Analysis of the bound water molecules shows that all of these four structures involve a water-mediated interaction with a conserved tyrosine in the bromodomains; RVX-208 makes a water-mediated interaction to Tyr97 in BRD4[BD1] and the equivalent Tyr386 in BRD2[BD2] through the 5-methoxy quinazolin-4-one oxygen. A second water mediated interaction is formed between BRD4[BD1] Gln85 and the 7-methoxy quinazolin-4-one oxygen, though an equivalent interaction is not found in the BRD2[BD2] complex. These water molecules have been previously recognized as structurally conserved among publicly available structures of bromodomains [29]. In summary, RVX-208 binds to each of the BET bromodomains in a similar manner, but quite distinct from how JQ-1 binds BET bromodomains.

### Thermodynamics of RVX-208 binding to BD1 versus BD2

To develop a fuller picture of why just two amino acid changes in the binding pocket between BD1 and BD2 could so substantially affect the binding affinity of RVX-208, we wanted to explore the enthalpy and entropy using isothermal titration calorimetry (ITC). Low concentrations of DMSO were used to decrease the influence of DMSO binding to the BET bromodomains on the thermodynamic parameters. This technique recapitulated the preference of RVX-208 for BD2 versus BD1 in both BRD2 and BRD4 observed previously (Table 5 and Figure S2). The data for both BRD2 and BRD4 indicate that in the conditions used, RVX-208 binding to BD1 induces a larger enthalpy gain than with BD2 combined with a loss of entropy. In contrast, the binding of RVX-208 to BD2 has a lower enthalpy gain but an increase in entropy.

**Table 5:** Thermodynamics parameters for binding of RVX-208 to purified BET bromodomains obtained from isothermal titration calorimetry.

Protein	*K*_d_, µM	Δ*H*, cal mol^−1^	Δ*S*, cal mol^−1^ K^−1^
BRD2[BD1]	16.9	−12670	−20.6
BRD2[BD2]	0.206	−4145	+16.7
BRD4[BD1]	8.93	−17040	−34.1
BRD4[BD2]	0.303	−6376	+8.44

### Determination of the role of different BRDs in driving ApoA-I induction

**Figure 9:**
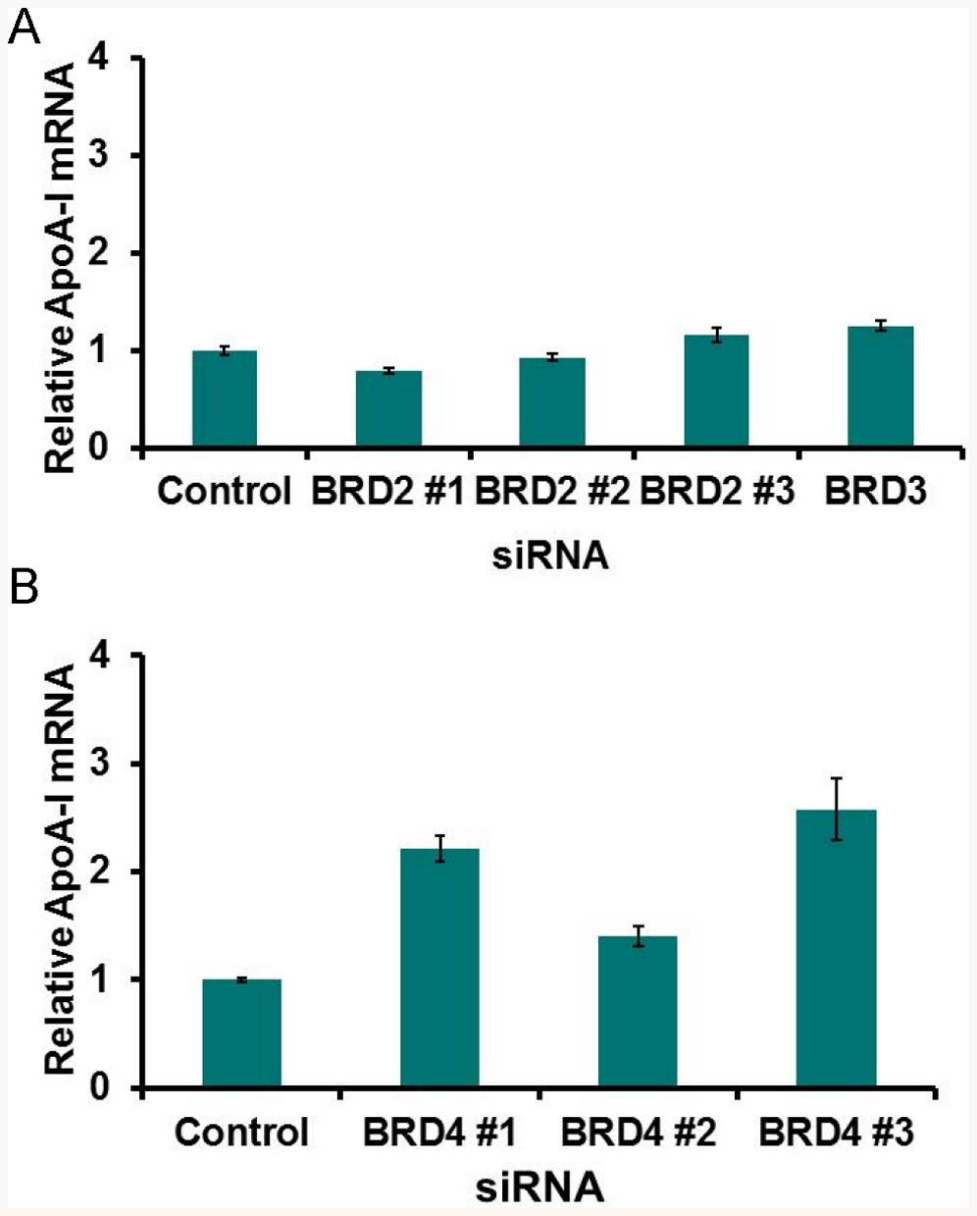
Effect of siRNAs directed toward different BET proteins on ApoA-I mRNA levels in Huh7 cells. A. Individual BRD2 and pooled BRD3 siRNAs. siRNAs were transfected for 48% DMSO. Protein knockdown was 56%, 42% and 44% for Brd2#1, #2 and #3 siRNAs and 86% for pooled Brd3 siRNAs. B. Individual BRD4 siRNAs. Huh-7 cells were transfected twice with individual siRNAs and after 72 h, cells were treated for 48 h with 0.1% DMSO. Protein knockdown was 86%, 83% and 93% for siRNAs 1, 2 and 3 respectively.

RVX-208 binds to the bromodomains of all three BET proteins expressed in human liver cells (BRD2, BRD3 and BRD4) leading to their dissociation from chromatin. To determine which of these proteins might drive ApoA-I expression, we treated Huh7 cells with siRNAs directed against all three BRDs, which might be considered to partially mimic the effect of RVX-208 in dissociating of BET proteins from chromatin. The results indicate that siRNAs directed to BRD4, but not BRD2 or BRD3, led to an increase in ApoA-I mRNA (Figure 9).

## Discussion

RVX-208, a compound that raises ApoA-I and HDL in humans, was originally discovered in a screen for inducers of ApoA-I mRNA in hepatocyte cell cultures [18], but its molecular target was not initially known. In this paper we have demonstrated that RVX-208 binds to the BET proteins which in turn induce ApoA-I expression via an epigenetic mechanism. The activity of BET proteins is mediated by the binding of their bromodomains to acetylated lysines of histones found in actively transcribing regions of chromatin [30]. Through this interaction, BET proteins recruit protein complexes to the site which in turn regulate transcription. By binding to the BET bromodomains and competing for the acetylated lysine binding site on BET proteins, RVX-208 causes the dissociation of BET and their associated proteins from chromatin, leading to altered transcription.

The results presented here contain several lines of evidence to firmly establish that this mechanism leads to induction of ApoA-I expression. First, we have shown that RVX-208 binds to BET bromodomains via several different techniques, including X-ray crystallography, isothermal calorimetry, thermal denaturation and HTRF-FRET and displaces bound acetylated histone peptide both in vitro and in cells. Consequently, RVX-208 now joins two other, distinct chemical scaffolds that bind to the bromodomains of BET proteins and induce ApoA-I [23], [24], [31], thus making it unlikely that the induction of ApoA-I is due to an off-target effect. Second, when variants of these scaffolds are studied, there is a correlation between binding affinity to the target and apoA-I induction (Fig. 4C and [31], [32]). Third, the use of siRNAs that suppress BRD4 expression induced ApoA-I expression in two different human hepatocyte cell lines: Huh7 cells in this study as well as in HepG2 cells [32]. In contrast BRD2 and BRD3 siRNAs had no effect. Together these data build a compelling argument that RVX-208 induction of ApoA-I gene transcription begins with its binding to BET proteins and that the dissociation of BRD4 in particular likely contributes to ApoA-I induction.

The co-crystal structure data of RVX-208 with a BD1 and BD2 domain demonstrate that RVX-208 binds in a unique manner compared to other published BET inhibitors. Furthermore, binding and calorimetric data indicate that this results in a preference for binding to BD2 over BD1 domains in the BET family. While the current data suggest that this difference may have an entropic component possibly due to a stacking interaction of the dimethyl-phenyl ring of RVX-208 with His433 not seen with Asp144 in BD1, we cannot rule out a pH dependent effect of the His433. The impact of differences in binding to BD1 and BD2 on biological activity is not known, although the energy of RVX-208 binding the two tandem domains in vitro appears to be an average of the two individual bromodomains.

Whereas the preceding studies detail the ApoA-I and resultant HDL raising effects of RVX-208, our data point to an additional activity of interest in treating atherosclerosis. BET antagonism using RVX-208 and JQ-1 regulates inflammatory cytokines by suppressing IL-6 but not TNFα in the U937 macrophage-like cell line. This is consistent with data indicating that treatment with siRNAs against the BET proteins also suppresses IL-6 in macrophages ([24]and data not shown). Hence the ability to potentially regulate both ApoA-I and macrophage cytokine activity, especially IL-6, appears to be an intrinsic property of compounds that bind to BET proteins. While abundant evidence supports favorable modulation of lipid levels having an anti-inflammatory effect, as seen in the studies of statins [33], these findings may suggest another benefit of RVX-208 in lowering IL-6. This cytokine is up-regulated in vulnerable atherosclerotic lesions [34] and higher levels of serum IL-6 are associated with an increased risk of coronary heart disease [35]. Furthermore, a variant of the IL-6 receptor has been genetically linked with increased likelihood of coronary heart disease [36], [37]. IL-6 in turn can stimulate levels of hsCRP, which is an additional risk factor for cardiovascular disease.

The interaction between BET proteins and the amino terminus of histones represents one of a growing class of protein-protein interactions (PPIs) that is amenable to small molecule drug discovery. Historically, these targets have been considered a challenge for small molecule inhibition due to the typically large surface areas involved at the interface between two proteins [38]. This is in contrast to the small, well defined pockets seen with traditional targets such as enzymes and G protein coupled receptors (GPCRs) that bind low molecular weight, natural ligands. Recently, a number of examples of inhibitors have emerged from targeting hotspots found in protein interaction surfaces that contribute most of the binding energy, and include both intracellular and extracellular targets. However, many of these inhibitors do not follow traditional Lipinski rules for what makes a successful small molecule, orally bioavailable drug, since they very often are of higher molecular weight [38]. The BET bromodomains have favorable properties in this regard since there are now several different, low molecular weight scaffolds that are orally available and have been shown to bind with affinities in the nM to µM range. It is likely that this is due in part to the limited size and shape of the protein interaction domain, and bodes well for the discovery of additional compounds that target BET bromodomains, and potentially other bromodomains.

The discovery of the molecular target of RVX-208 emphasizes the power of using phenotypic screens for the identification of new drugs [39]. Through the years many drugs have been discovered in this fashion such as cyclosporine [40] and rapamycin [41], and it was only subsequently that their molecular target and mechanism of action were defined [42], [43]. There remain several examples today where the specific molecular target of marketed drugs is not fully understood, such as niacin [44], BG-12 [45] and lenalidomide [46]. However, there is significant value in identifying a molecular target for these compounds, since it enables the development of mechanism based biomarkers to evaluate target engagement and pathway modulation as part of pharmacokinetic/pharmacodynamic modeling, and to potentially identify the patients who will most benefit from the therapy. RVX-208 in this respect is somewhat unique since it has two well-defined biomarkers, ApoA-I and HDL-C, as its mechanistic readouts, which were key drivers in determining the screening strategy by which it was discovered, and have been a critical part of its clinical development. Nevertheless, the identification of a molecular target will enable additional research into the mechanism of action of RVX-208 and its activity in the clinic.

The finding that RVX-208 regulates transcription through an epigenetic mechanism mediated by the BET proteins further extends the promise of epigenetics as a potential source of new therapies to address several unmet medical needs. The first epigenetically targeted therapies to enter clinical practice include the histone deacetylase (HDAC) and DNA methyltransferase inhibitors [47], which have been approved for the treatment of cutaneous T cell lymphoma and myelodysplastic syndromes respectively, and are being actively investigated for other cancers. However, since the discovery of these compounds, and aided by the revolution in genomics technologies, there has been a rapid increase the discovery of new families of proteins that epigenetically regulate transcription in the cell. Many of these regulate the process of adding or removing posttranslational modifications such as acetylation, methylation, and ubiquitination to the protein components of chromatin such as histones, a process that has been likened to “writing” and “erasing” [48], [49]. This code of modifications is then “read” by proteins such as the BET proteins, which in turn recruit additional proteins that regulate the process of transcription. With HDAC inhibitors, sirtuin activators and inhibitors and methyltransferase inhibitors already approved or in clinical development, there are examples of drugs in development that target the writing and erasing steps. Now that we have discovered that RVX-208 blocks the reading step mediated by the BET proteins, we have a further category of the epigenetic process that is potentially amenable to drug intervention. RVX-208 has been in the clinic for many years and has been administered to over 800 patients [18], [19], [20]. More recently, two other BET inhibitors have entered the clinic for the treatment of cancer [50], [51]. The combined clinical experience with RVX-208 and other BET inhibitors will help determine the utility of this class of molecules in various medical indications.

## Supporting Information

Figure S1 **Thermal denaturation of RVX-208 binding to BET bromodomains.** 5 µM of purified bromodomain protein and SYPRO® Orange were heated in the presence of 100 µM RVX-208, JQ1 or buffer control, and the fluorescence measured as a function of temperature.
(TIF)

Figure S2 **Isothermal calorimetry of RVX-208 binding BRD2 and BRD4 bromodomains.** Individual bromodomains at 150 µM, BRD2[BD1], BRD2[BD2], BRD4[BD1], or BRD4[BD2] were titrated into 10 µM RVX-208 and an integrated curve fit to data with background correction and modeled for single site binding.
(TIF)
