# Supplementary figures and images for: RVX-208, an Inducer of ApoA-I in Humans, Is a BET Bromodomain Antagonist
Source: PLoS One. 2013 Dec 31;8(12):e83190. doi: 10.1371/journal.pone.0083190 (PMC3877016; doi:10.1371/journal.pone.0083190)

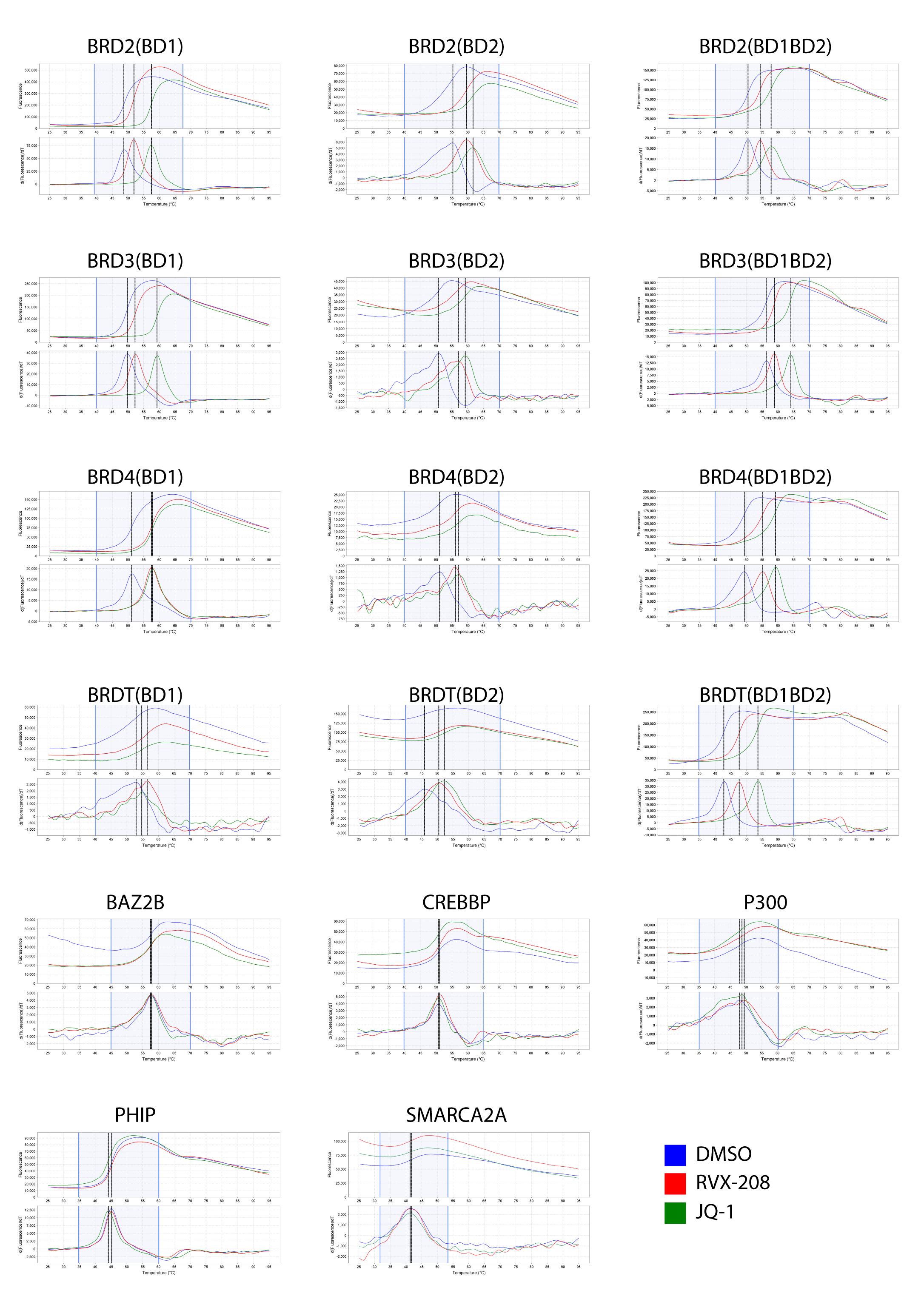

Supplement: Figure S1 — Thermal denaturation of RVX-208 binding to BET bromodomains. 5 µM of purified bromodomain protein and SYPRO® Orange were heated in the presence of 100 µM RVX-208, JQ1 or buffer control, and the fluorescence measured as a function of temperature. (TIF) [file pone.0083190.s001.tif]

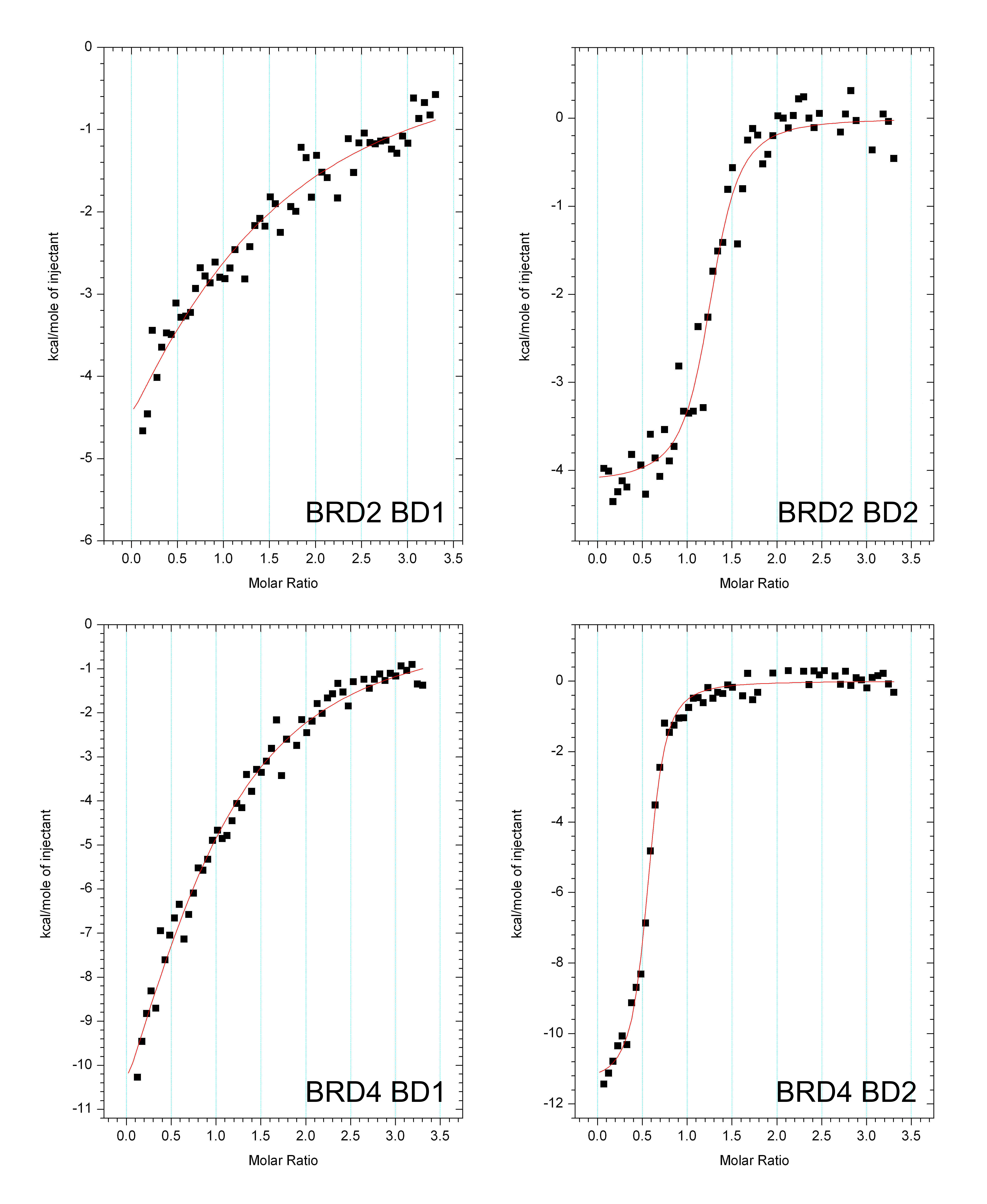

Supplement: Figure S2 — Isothermal calorimetry of RVX-208 binding BRD2 and BRD4 bromodomains. Individual bromodomains at 150 µM, BRD2[BD1], BRD2[BD2], BRD4[BD1], or BRD4[BD2] were titrated into 10 µM RVX-208 and an integrated curve fit to data with background correction and modeled for single site binding. (TIF) [file pone.0083190.s002.tiff]
